# An updated system for categorising and reporting unintended incidents in radiotherapy at a national level

**DOI:** 10.2340/1651-226X.2026.45699

**Published:** 2026-05-11

**Authors:** Kirsten L. Jakobsen, Annette R. Jakobsen, Karina L. Gottlieb, Martin Berg, Harald Spejlborg, Susan B.N. Biancardo, Bob Smulders, Heidi S. Rønde

**Affiliations:** a Department of Oncology and Palliative Care, University Hospital Region Zeeland, Næstved, Denmark; b Department of Medical Physics, Department of Oncology, Aalborg University Hospital, Aalborg, Denmark; c Laboratory of Radiation Physics, Department of Oncology, Odense University Hospital, Odense, Denmark; d Department of Medical Physics, Department of Oncology, Vejle Hospital, Lillebaelt Hospital – University Hospital of Southern Denmark, Vejle, Denmark; e Department of Medical Physics, Aarhus University Hospital, Aarhus, Denmark; f Department of Oncology, Copenhagen University Hospital – Herlev and Gentofte, Denmark; g Department of Oncology, Copenhagen University Hospital, Rigshospitalet, Copenhagen, Denmark; h Danish Centre for Particle Therapy, Aarhus University Hospital, Aarhus, Denmark

**Keywords:** Radiotherapy, patient safety, unintended events, risk management

## Abstract

**Background and purpose:**

Radiotherapy is a complex, multistage process that involves collaboration across professional disciplines (radiographers working in pre-treatment preparation, physicians, physicists, radiation therapists during planning and treatment. While severe errors are uncommon, they can have profound consequences for patients. The Danish Society for Medical Physics supports a Special Interest Group dedicated to improving patient safety by analysing unintended events (UEs) and near misses in radiotherapy. Here we report on the work conducted with an updated system for categorising UEs in radiotherapy in Denmark.

**Material and methods:**

An updated reporting system based on the English Radiotherapy Pathway Coding (RPC) system has been taken into use in 2025. This system, including the use of a Danish system that ranks the UEs from the patients perspective, called Patient Centred Coding, is outlined and UEs reported in 2025 are summarised.

**Results:**

Radiotherapy is generally a safe treatment modality, a conclusion further supported by our results from 2025. In 2025 only 339 UEs were reported, 315 of these with low risk and 24 with medium risks. The lesson learned is that the main focus in order to prevent the most harmful UEs should be on the delineation and the planning process.

**Interpretation:**

Based on our experience from this work, we recommend the development of a national system for categorising and ranking UEs. We further recommend establishing a national working group that meets regularly and focuses on UEs at a national level.

## Introduction

Radiotherapy is one of the most widely applied treatment modalities for cancer both as a primary intervention and as adjuvant therapy. In Denmark approximately 47,000 individuals are diagnosed with cancer every year and an estimated 50% receive at least one course of radiotherapy [1]. Despite its complexity and the involvement of multiple professional disciplines (radiographers (pre-treatment preparation), physicians, physicists and radiation therapists (RTTs), radiotherapy is generally regarded as a safe treatment modality, with a low number of reported unintended events (UEs) [2]. While most UEs in radiotherapy have minor effects and do no harm to the patients involved, there still may remain a risk for UEs leading to serious patient consequences. Especially those UEs related to, for example, malfunctions of equipment and systematic human errors can potentially affect the clinical outcome of numerous patients.

Given these considerations, systematic efforts to ensure patient safety are essential. In Denmark, this work is supported by the ‘Danish Patient Safety Database’ (DPSD) [3], which provides an established infrastructure for reporting UEs across the entire healthcare system in general as confirmed in the Danish health act [4]. However, DPSD is a general healthcare reporting system, which does not let users categorise UEs into radiotherapy-specific sub-categories. This makes systematic monitoring, reporting and comparisons within radiotherapy difficult. Thus, analysis of patient safety within Danish radiotherapy calls for an additional system for categorisation and description of UEs. National systematic reporting enables continuous monitoring of safety issues across Danish radiotherapy centres and creates the empirical basis necessary for analysing error patterns. This approach allows for the identification of system vulnerabilities and facilitates the implementation of targeted preventive measures. Strengthening the reporting culture and ensuring consistent documentation of UEs are therefore crucial steps towards reducing the frequency of such events and maintaining a high level of patient safety in radiotherapy.

In 2012, the Danish Cancer Society established a working group to evaluate the safety of radiotherapy nationally. A literature review and international comparison highlighted the Radiotherapy Pathway Coding system (RPC) developed in the United Kingdom [5], which was subsequently adapted for use in the Danish context. Patient representatives participating in the working group identified a need to complement the RPC system with a Patient Centered Code (PCC) framework.

As a direct outcome of the 2012 national meeting, a Special Interest Group (SIG) on Patient Safety in radiotherapy was established under the auspices of the Danish Society for Medical Physics (DSMF) in 2017. Although case sharing continued, the group’s activity over the subsequent years was intermittent, with limited engagement and irregular, informally structured meetings. In 2022, the remaining active members undertook a structured revitalisation of the initiative, formalising its governance, establishing terms of reference, and reconstituting the SIG into its current form. Since the SIG was established under DSMF (whose members comprise solely physicists) and today still reports to the DSMF board, the group has historically not been multidisciplinary. Even so, the SIG has now been expanded to include both RTTs, radiographers and medical physicists; a radiation oncologist representative has not yet been recruited to the national group although physicians are active in all local groups. As documented here there is a long tradition and legal requirement in Denmark for reporting UEs. In the later years the importance of this is recognised globally and more and more countries are working in the same direction [6–9].

The SIG has developed an updated RPC system tailored to current radiotherapy-practices in Denmark. The result of this work is presented in this article along with the PCC scoring exemplified by the UEs from 2025.

## Material and methods

DPSD is a system that surveys the safety of the total healthcare system in Denmark. Employees are obliged to report both actual and potential (near misses) serious and lethal UEs, including UEs with a learning potential. This can be done anonymously. From DPSD the case is transferred to the specific department of origin. Each radiotherapy centre has a multidisciplinary panel that reviews the UEs and ensures learning from relevant cases. Importantly, the DPSD is a non-sanctioning environment.

A UE report in DPSD contains a free text description and a risk evaluation. Thus, all UEs and near misses are registered with an actual consequence and a potential consequence. These are then merged to the combined risk of the event (Table 1).

**Table 1:** Risk scoring when reporting UEs in DPSD.

Actual consequence		Potential consequence		Combined risk
None/unknown	+	None	=	Low
None/unknown	+	Minor/Moderate	=	Low
None/unknown	+	Serious	=	Medium
None/unknown	+	Lethal	=	High
Minor/moderate	+	Minor/moderate	=	Low
Minor/moderate	+	Serious	=	Medium
Minor/moderate	+	Lethal	=	High
Serious	+	Serious	=	High
Serious	+	Lethal	=	High
Lethal	+	N/A	=	High

The first and second columns refer to the actual and potential consequence of the UE under consideration. The combination of these two leads into a combined risk shown in the third column. UE: unintended events; DPSD: Danish Patient Safety Database.

While reviewing the UE categories provided in the RPC system, it became evident that the original set of 21 UE origins was no longer up-to-date. A closer analysis of the current radiotherapy workflow resulted in an updated RPC system with 14 categories (Table 2). These range from room design to handling of side effects. All 14 categories have been divided into sub-categories giving a total of 150 sub-categories (See Supplementary Material). The subdivision enables a more precise analysis of where in the processes UEs occur and focuses the work towards preventing UEs even more. Naturally, such subgroup analyses are only feasible when sufficient data are available to ensure statistical significance.

**Table 2:** The Danish RPC-coding anno 2025 consists of 14 items.

Process code	Activity description
0	Room design
1	External visitation and referral
2	Internal visitation and referral
3	Doctors’ appointment
4	Booking process (pretreatment and treatment)
5	Fixation before and at scanner
6	Scanning
7	Image handling
8	Delineation
9	Dose planning
10	Independent checks
11	Treatment
12	Medication/concomitant treatment
13	Handling of side effects

RPC: Radiotherapy Pathway Coding.

The PCC scores the potential risk for the patient, with low scores corresponding to high perceived risk. The most severe effect for a patient would be an impact of the UE on the treated volume. This would affect the tumour control probability, risk of side effects, or both. A UE affecting the follow-up would be of less harm as seen from the patient perspective, leading to a higher PCC score. The framework of the PCC is outlined in Table 3.

**Table 3:** The Danish patient centered code (PCC) system consists of 10 items.

Item	Impact area
1	Treatment volume
2	Dose/fractionation
3	Waiting time/treatment delay
4	Concomitant treatment
5	Technical equipment
6	Independent checks
7	Communication/documentation
8	Patient experience communication/documentation
9	Follow-up
10	Other

The lower the number, the greater the effect on the patient as seen from the patient’s perspective.

## Results

Anonymised UE data including PCC and RPC, patient actual and potential harm, and combined overall risk from each of the eight Danish radiotherapy centres reported in 2025, where more than 240,000 external radiotherapy fractions were given, were collected. In total 339 UEs were reported: 315 incidents with low and 24 incidents with medium combined patient risk. This is comparable to the numbers in 2024: 315 UEs, 299 incidents with low risk, 15 with medium risk and 1 with high risk. As we have changed the RPC system the categorisation cannot be compared directly between the 2 years.

**Figure 1:**
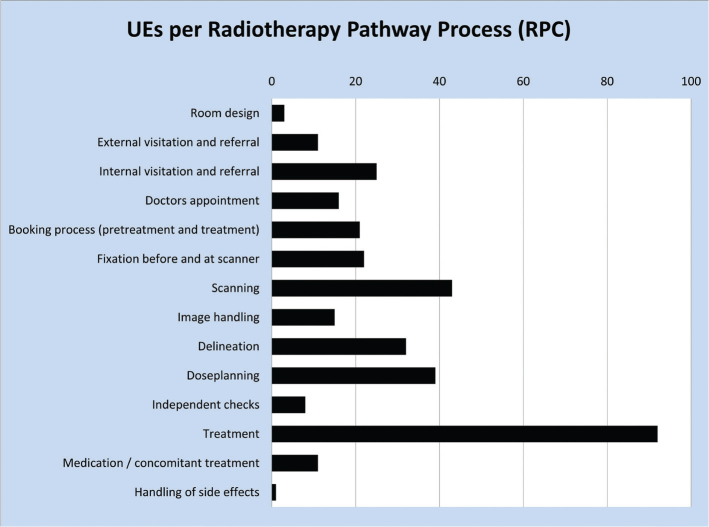
Unintended events categorised in RPC. RPC: Radiotherapy Pathway Coding.

Figure 1 shows the distribution of UEs across the various steps in the radiotherapy workflow. Events most frequently (27%) originate in the treatment process, which is likely due to the multiple number of fractions compared to the single instance of planning and simulation stages. However, a considerable number of UEs are also reported during scanning (13%), delineation (9%) and treatment planning (12%).

**Figure 2:**
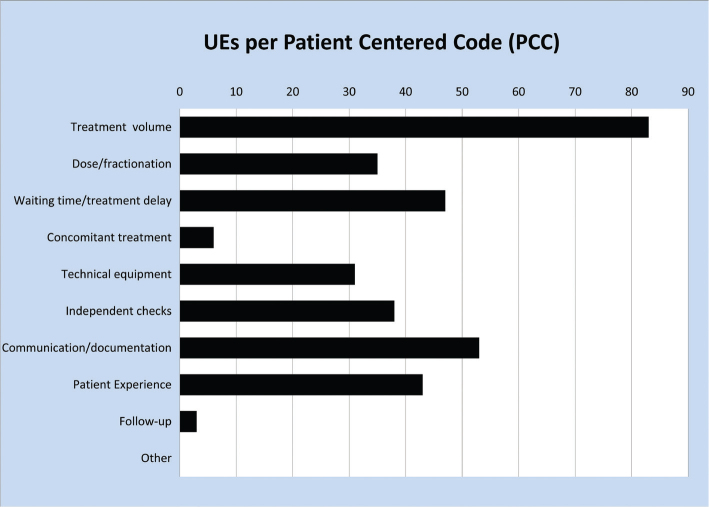
Unintended events categorised in PCC. PCC: patient centered code.

**Figure 3:**
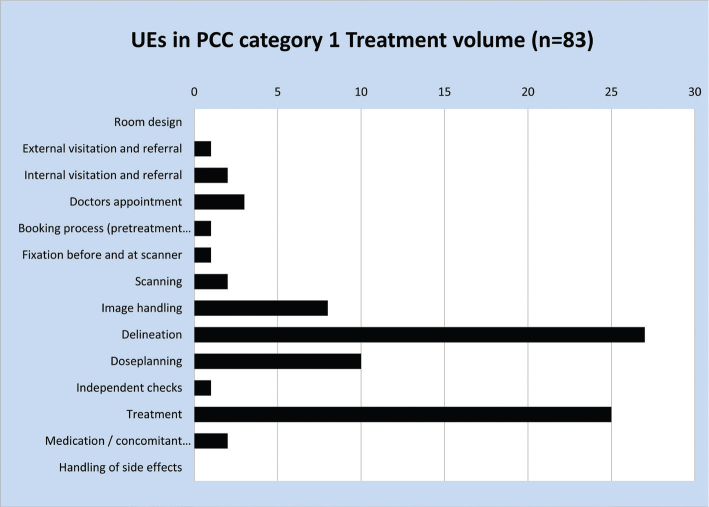
Origin in the RPC system for unintended events in PCC category 1 (Treatment volume). PCC: patient centered code; RPC: Radiotherapy Pathway Coding.

While Figure 1 demonstrates in which workflow steps there may be room for improvements, it yields no information on the severity of the UEs as seen from a patient perspective. This information is provided by the PCC data. Figure 2 shows the ranking of the UEs in the PCC system. It is seen that the largest number of UEs (*n* = 83, 24%) is found in category 1 (treatment volume), which are also the UEs carrying the highest risk seen from the patients’ point of view. In order to find the RPC categories where UEs with the most harmful potential originate, all UEs with PCC category 1 were categorised by the RPC system. Figure 3 demonstrates that most UEs affecting treatment volume originate in delineation (*n* = 27, 33%) and treatment (*n* = 25, 30%).

## Discussion and conclusion

Learning from reported UEs is essential within radiotherapy, where errors can lead to serious harm to patients. A prerequisite to learning is a system supporting the reporting itself, a meaningful categorisation of the UEs, and last but not least presentation of the collected data to all staff members contributing to the radiotherapy course. In Denmark the reporting system was already present, but a standardised, contemporary national radiotherapy-specific system for categorising UEs was missing. This has now been developed and implemented.

During the analysis of the UEs in 2025 the SIG had access to UEs on an institutional level. In order to maintain the anonymity of the centres only the collective results are presented in this paper.

The UEs and their distribution patterns were found to be very similar across the eight Danish departments, even though both their software (Oncology Information Systems, Treatment Planning Systems) and hardware (computer tomography [CT], magnetic resonance imaging [MRI], linear accelerators) differ. This was very encouraging since it supports the idea that clinics can learn from each other in spite of differences in their technological set-up. The PCC and updated RPC categorisations generate more detailed knowledge on where in the radiotherapy process UEs occur and how severe they are. This makes it possible to focus efforts where it yields the highest value, for example, on multiple similar UEs or UEs with high impact on patient safety.

No UEs with a high risk score were reported, indicating that no events with serious or lethal consequences occurred in 2025. This suggests that radiotherapy in Denmark remains a safe treatment modality. The error rate of 1.4 per 1000 treatments is comparable to the rate of 1.3 per 1000 found by Smith et al. [10].

Based on the reported distribution of UEs (Figures 1 and 2) it seems relevant to focus efforts around the pre-treatment workflow steps; delineation, prescription and dose planning. Errors in these processes may systematically influence the entire treatment, as opposed to an error occurring only once in the course of multiple treatment fractions. By having a national system to report UEs, improvements are based on statistics, and not ‘feelings’, and a common language has evolved. Experience has shown that it is important to maintain regular meetings with participants from across the country; otherwise these common agreements tend to evolve in different directions.

One of the limitations of any reporting system is the risk of under-reporting of UEs. In Denmark this work is supported by the law [11]. This means local management supports allocating resources to the task of collecting and disseminating the data. Furthermore, a non- sanctioning system with optional anonymous reporting promotes higher reporting rates and leads to stronger organisational learning. A system built as described in this work is known to increase the numbers of reported UEs [12].

In general, there is an expected under-reporting primarily on the less severe UEs and on ‘busy days’. Since the employee who identifies a UE is also the one responsible for reporting it, RTTs report more UEs than other staff members. This is partly a consequence of the large number of tasks they perform throughout the treatment course. It is also related to the numerous procedures that take place before RTTs encounter the patient for treatment. At each of these stages, there is a potential for a UE to occur [13].

Literature can be found on accidental exposures and overall design of an Incident Learning System [14–17]. In a busy clinic an easy and fast system for registration of the UEs is essential. To maintain the commitment to continuous reporting it is relevant to have a national system to obtain relevant feedback. Contemporary radiotherapy is more complex than ever with lots of tasks and information going back and forth between different professional groups – based on our work we believe the combination of an updated RPC and PCC highlights the areas of greatest impact for patient safety.

Over time when data mature more, comprehensive analysis will be made regarding treatment quality on a national and institutional level. The SIG plans to ask all members to score the same UEs in order to explore the consistency across centres. To ensure the best learning for patient safety it is highly recommended that all professions are represented in the local UE panels.

To conclude, the updated Danish system of Patient Centered Code and RPC to report and track (systematic) UEs is recommended. Furthermore, it is strongly recommended for each country to align RPC with local workflow to obtain local site-specific insights into areas requiring improvement.

## Supplementary Material



## Data Availability

Data can be shared upon reasonable request.
