# Supplementary material for: An updated system for categorising and reporting unintended incidents in radiotherapy at a national level
Source: Acta Oncol. 2026 May 11;65:45699. doi: 10.2340/1651-226X.2026.45699 (PMC13169485; doi:10.2340/1651-226X.2026.45699)
Supplement: Supplementary file 1 [file AO-65-45699-s1.pdf]

Supplementary material has been published as submitted. It has not been copyedited, or typeset by Acta Oncologica

Classification of Unintended Events in Radiotherapy

Modalities: Photons, electrons, protons, brachytherapy, and superficial radiotherapy
Scope: From referral to final treatment fraction

Step	Area	Proces
0	Facilities and Equipment	A. Bunker/facility, Equipment, Dosimetry, Modelling
1	External Referral and Triage (Non-oncology setting)	A. Patient identification<br>B. Missing/incorrect documentation<br>C. Communication (internal/external)<br>D. Incorrect referral<br>E. Missing documentation of prior treatment<br>F. MRI contraindication screening<br>G. Other
2	Internal Referral and Triage (Oncology setting)	A. Patient identification<br>B. Missing/incorrect documentation<br>C. Communication (internal/external)<br>D. Diagnosis<br>E. Treatment site (including laterality)<br>F. Dose/fractionation<br>G. Contraindications (previous treatment, pregnancy, pacemaker, implants, etc.)<br>H. Other
3	Medical Consultation	A. Patient identification<br>B. Missing/incorrect documentation<br>C. Communication (internal/external)<br>D. Patient communication<br>E. Medication dispensing<br>F. Consent<br>G. Protocol information<br>H. MRI contraindication screening<br>I. Other
4	Booking of Radiotherapy Course	A. Patient identification<br>B. Missing/incorrect documentation<br>C. Communication (internal/external)<br>D. Scheduling (appointments, dates, attendance)<br>E. Patient communication<br>F. Treatment postponement<br>G. Other
5	Positioning and Immobilisation	A. Patient identification<br>B. Missing/incorrect documentation<br>C. Communication (internal/external)<br>D. Positioning<br>E. Immobilisation devices

		F. Markings on immobilisation devices<br>G. Bolus<br>H. Patient markings<br>I. Other
6	Imaging (incl gating and 4D)	A. Patient identification<br>B. Missing/incorrect documentation<br>C. Communication (internal/external)<br>D. Patient communication<br>E. Imaging protocol<br>F. Image quality (artefacts)<br>G. Contrast<br>H. MRI contraindications<br>I. Reconstruction<br>J. Scan length/FoV<br>K. 4D/gating/breath hold<br>L. Bladder filling<br>M. Rectum/bowel filling<br>N. Ventricular filling<br>O. Other
7	Image Handling	A. Patient identification<br>B. Missing/incorrect documentation<br>C. Communication (internal/external)<br>D. Correct scan selection<br>E. Registration/fusion (current scans)<br>F. Registration/fusion (previous scans and dose)<br>G. 4D/gating handling<br>H. Other
8	Contouring (incl OOI, targets, AI, etc)	A. Patient identification<br>B. Missing/incorrect documentation<br>C. Communication (internal/external)<br>D. Correct scan selection<br>E. Target volumes<br>F. Organs of interest (OOI)<br>G. Artificial intelligence<br>H. Bolus<br>I. Naming<br>J. Margins (including 4D)<br>K. Previous treatment considerations<br>L. Documentation (compromises)<br>M. Other
9	Treatment Planning	A. Patient identification<br>B. Missing/incorrect documentation<br>C. Communication (internal/external)<br>D. Immobilisation considerations<br>E. Positioning considerations<br>F. Previous treatment considerations<br>G. Artificial intelligence<br>H. Fractionation

		I. Dose to target<br>J. Dose to OOI<br>K. Modality/energy<br>L. Bolus<br>M. Technique/algorithm selection<br>N. Software errors/bugs/issues<br>O. Documentation (compromises, plan selection)<br>P. Other
10	Independent Verification (incl measurements)	A. Patient identification<br>B. Missing/incorrect documentation<br>C. Communication (internal/external)<br>D. Independent dose calculation<br>E. Independent plan check<br>F. Not performed<br>G. Other
11	Medication and Concomitant Treatment	A. Patient identification<br>B. Missing/incorrect documentation<br>C. Communication (internal/external)<br>D. RTT check before treatment<br>E. Positioning<br>F. Immobilisation devices<br>G. Markings on devices<br>H. Bolus<br>I. Patient markings<br>J. 4D/gating/breath hold<br>K. Image import<br>L. Image acquisition (CBCT, MV, kV)<br>M. Image matching – target (online)<br>N. Image matching – OOI (online)<br>O. Anatomical changes (online)<br>P. Collision<br>Q. Equipment failure<br>R. Image matching – target (offline)<br>S. Image matching – OOI (offline)<br>T. Anatomical changes (offline)<br>U. Adaptation (offline)<br>V. Adaptation (online)<br>W. Patient communication<br>X. Shielding/blocking<br>Y. Other
12	Medication and Concomitant Treatment	A. Patient identification<br>B. Missing/incorrect documentation<br>C. Communication (internal/external)<br>D. Other
13	Management of Side Effects	A. Patient identification<br>B. Missing/incorrect documentation

		C. Communication (internal/external)<br>D. Other

Comments

One code should be selected per unintended event (e.g., “11S”). Independent events for the same patient should be recorded separately.
